# Technology and engineering test of filling goaf with coal gangue slurry

**DOI:** 10.1038/s41598-023-47621-8

**Published:** 2023-11-23

**Authors:** Shengrong Xie, Hao Pan, Wenzhe Gu, Lei Zhu, Dong Yue, Dongdong Chen, Tianqi Song, Zaisheng Jiang

**Affiliations:** 1 School of Energy and Mining Engineering, China University of Mining and Technology-Beijing, Beijing, 100083 China; 2 Chinacoal Research Institute Co. Ltd, Xian, 710054 China; 3 Yulin Yanghoupan Mining Co. Ltd, Yulin, 719000 China

**Keywords:** Environmental impact, Engineering

## Abstract

Based on the greening and low interference disposal requirements of coal gangue in high-yield and high-efficiency mines in Inner Mongolia and Shaanxi, and by integrating the existing theories and technologies such as underground filling technology of coal gangue, mine yellow mud grouting technology, and the evolution law of mining overburden fractures, a technology of filling goaf with coal gangue slurry for green disposal of coal gangue is proposed. The principle and technical framework of the technology of filling goaf with coal gangue slurry are clarified. This paper expounds on the technological process of technology of filling goaf with coal gangue slurry, establishes three types of slurry filling systems, such as centralized ground layout, ground and underground coordinated layout, and centralized underground layout, and constructs three slurry filling methods, including low-level grouting, adjacent level grouting, and high-level grouting, forming seven kinds of technology models of filling goaf with coal gangue slurry, including ground centralized pulping + high-level grouting, ground centralized pulping + adjacent grouting, ground centralized pulping + low level grouting, ground and underground coordinated pulping + adjacent grouting, ground and underground coordinated pulping + low level grouting, underground centralized pulping + adjacent grouting, and underground centralized pulping + low level grouting, and gives the selection process of technology models of filling goaf with coal gangue slurry. Based on the different conditions and requirements of Haidaze Coal Mine and Huangling No. 2 Coal Mine, engineering tests were carried out on two different technology modes, namely, ground and underground coordinated pulping + low level grouting and ground and underground coordinated pulping + adjacent grouting, based on simulation tests of low level grouting and adjacent grouting. The tests prove the feasibility of filling goaf with coal gangue slurry and explore the way for the theoretical research of the technology of filling goaf with coal gangue slurry and greening and low interference disposal of coal gangue.

## Introduction

Coal gangue refers to the carbon-containing solid waste discharged from the coal mine during the development and excavation, coal mining, raw coal washing, and other production processes. It is mainly composed of a mixture of driving, washing, sorting gangue, hand-picked gangue, and white gangue outside the coal series stacked with coal gangue. It is usually discharged to the ground as solid waste, forming a unique gangue hill in the mining area. According to incomplete statistics, the total amount of coal gangue stockpiled in China exceeds 7 billion tons, forming about 1500–1700 gangue hills, and the annual emissions increase by 300–350 million tons^1^. The discharge of a large amount of coal gangue is bound to cause ecological and environmental problems such as land occupation and water pollution^2^.

Under the requirements of energy supply guarantee and high-quality development of the coal industry, large-scale and efficient comprehensive utilization of coal gangue will be the only way for large coal enterprises to achieve green and sustainable development, and also one of the important ways to implement the dual carbon goals. The long-term theoretical research and practice have proved that coal gangue underground filling is the most reliable, thorough, and lowest cost of supervision of green disposal^3^. China's underground filling technology of coal gangue has a history of decades of development. After exploring generations of mining predecessors, three underground filling technologies of coal gangue, such as solid filling, paste filling, and overburden separation grouting filling, have gradually formed^4–7^. The solid filling is to install professional filling hydraulic support at the stope face, and then use the rear scraper with a gangue chute behind the hydraulic support to drop the material, and use the tamping mechanism at the back of the hydraulic support to compact the fallen gangue, to support the stope roof to weaken the movement of overburden, and realize the dual role of surface subsidence reduction and coal gangue disposal^8, 9^. Paste filling makes paste slurry from coal gangue, cement, fly ash, and water. Then use the filling pump or self-weight to transport the paste to the underground goaf through the pipeline, and then replace the coal resources to support the roof of the stope to control the movement of the rock stratum and dispose of part of the gangue^10, 11^. Grouting filling of the overburden separation zone is to make gangue slurry from coal gangue and water. Then use the grouting pump to fill the gangue slurry to the separation area under the vital overburden layer of the stope through the ground drilling to prevent the critical layer from breaking. Moreover, it can control the movement of rock stratum and surface and realize the underground filling of coal gangue^12–14^.

Under the background of double carbon, starting from the requirements of green disposal of coal gangue, the existing three kinds of coal gangue of underground filling methods can realize the green disposal of coal gangue and have been widely used. However, they affect mine production, investment cost, process complexity, and applicability. Both solid filling and paste filling need to add the new working face. Its high investment and complex process flow make it mainly used in "three under and one above" coal mining, taking into account underground filling coal gangue, and the scale of paste filling and disposal of coal gangue is relatively limited. Identification and determination of the separation zone of mining overburden strata in the grouting filling of overburden strata is the core problem of the filling technology. The coal seam mining area needs to have a certain thickness of bedrock to restrict the large-scale promotion of the technology^15–18^. According to the above analysis, based on the disposal requirements of green coal gangue with low interference in high-yield and high-efficiency mines in Inner Mongolia and Shaanxi, the technology of filling goaf with coal gangue slurry is proposed by combining the existing filling technology of coal gangue underground, the grouting technology of mine yellow mud and the evolution law of mining-induced overburden cracks^19–27^. The principle and process flow of technology of filling goaf with coal gangue slurry are clarified, and the slurry filling system and filling method are constructed. The technology mode of filling goaf with coal gangue slurry under different conditions is formed. The simulation test and engineering tests of filling goaf with coal gangue slurry in Dahaize Coal Mine and Huangling No.2 Coal Mine are described in detail. It explores the road for the theoretical research of the technology of filling goaf with coal gangue slurry and greening and low-interference disposal of coal gangue.

## Technology of filling goaf with coal gangue slurry

### The technical framework of filling goaf with coal gangue slurry

The technology of filling goaf with coal gangue slurry is to make gangue into granular aggregate with a specific particle size gradation by crushing, screening, and other technical means, mixing and stirring it with water in proportion to make gangue slurry with a specific concentration. Then the filling pump is used to fill the gangue slurry to the goaf by pipeline transportation to finally realize the coal gangue is green disposal without affecting the coal mine's average production.

**Figure 1:**
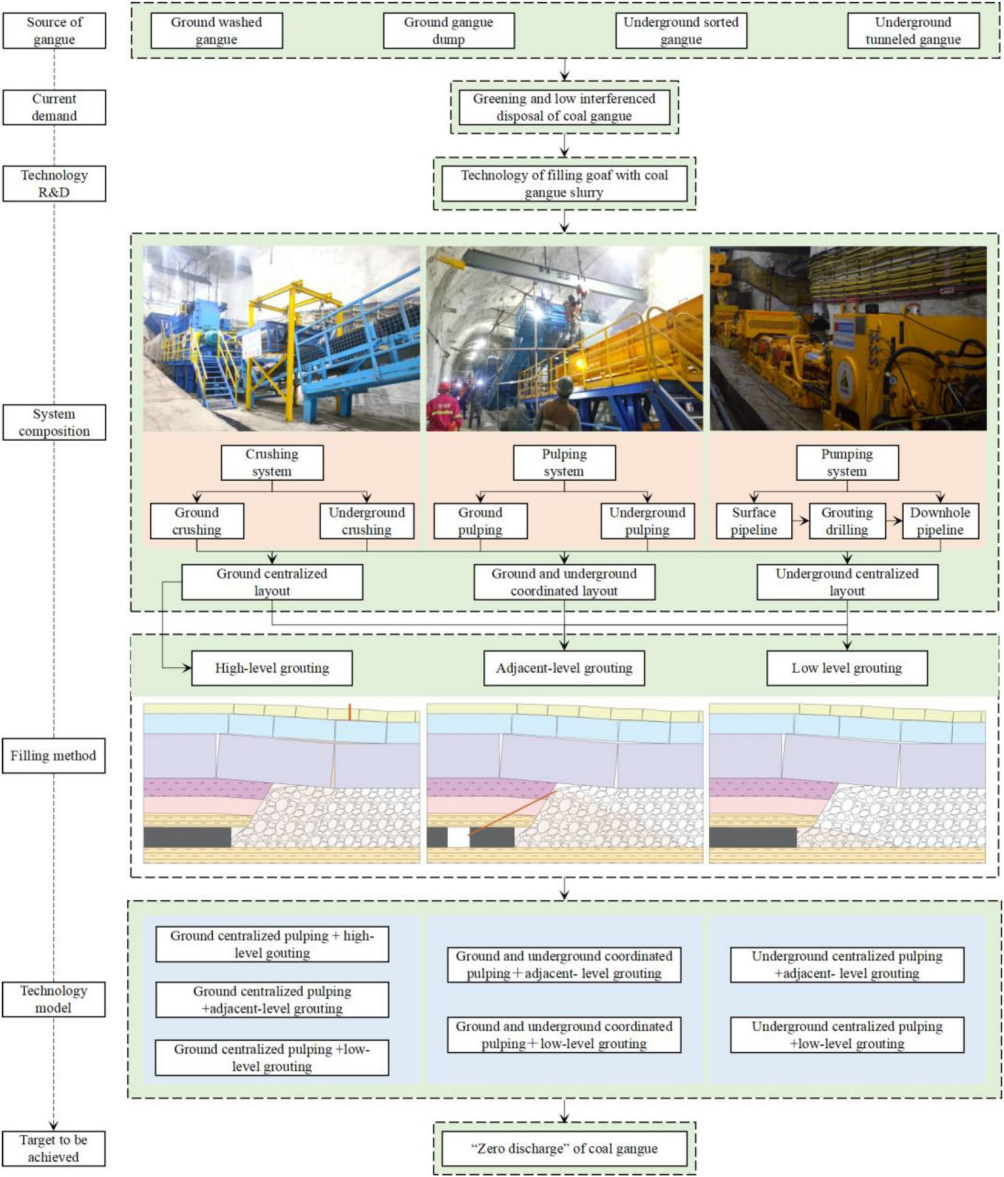
The technical framework of filling goaf with coal gangue slurry.

The technology of filling goaf with coal gangue slurry consists of three parts: crushing system, pulping system, and pumping system; each system is connected and coordinated. In combination with the source of gangue, plant location, and roadway layout, three system layout forms are formed: ground centralized layout, ground and underground coordinated layout, and underground centralized layout. According to the pipeline laying path at the end and the location of the target goaf, three filling methods are formed, including low-level grouting filling, adjacent grouting filling, and high-level grouting filling. The good connection between the system layout and the filling method is realized by optimizing the spatial layout of the relevant positions or the roadway chambers of crushing, pulping, and pumping. The optimal organization of work efficiency is achieved based on the coordination and matching of filling and coal mining technology in time. So seven adaptive technology models of filling goaf with coal gangue slurry are constructed, and the matching of gangue output, goaf space, and technology model is considered as a whole to finally achieve the goal of “zero discharge” of coal gangue, as shown in Fig. 1.

### Process of filling goaf with coal gangue slurry

**Figure 2:**
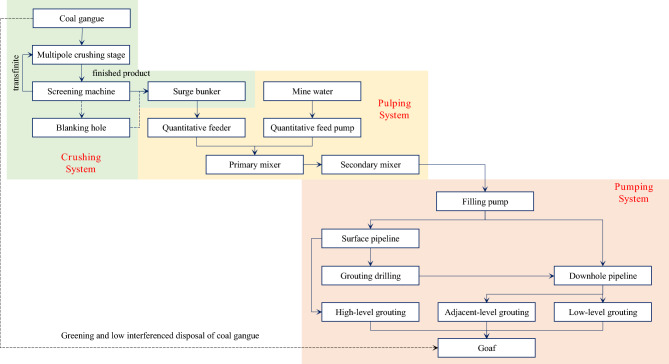
Process of filling goaf with coal gangue slurry.

The process of filling goaf with coal gangue slurry is shown in Fig. 2. Specifically: the coal gangue is crushed by a multi-stage crusher and enters the screening machine for screening, the finished particle aggregate under the screen is transferred to the finished product surge buffer, and the large particle aggregate on the screen enters the crusher through the return belt for further crushing. When filling the slurry, the quantitative feeder and the quantitative feed pump transport the finished particle aggregate and mine water to the primary mixer in a specific proportion for pulping. The qualified slurry overflows to the secondary mixer for buffer mixing and then flows automatically to the receiving hopper in the filling pump. Finally, after being pressurized by the filling pump, the gangue slurry in the receiving hopper is filled to the separation zone, fracture zone, or caving zone of the goaf using high-level grouting, adjacent-level grouting, or low-level grouting through the pipeline to realize the green and low interference disposal of coal gangue.

### Filling system and filling method of coal gangue slurry

#### System layout of filling goaf with coal gangue slurry

The technology system of filling goaf with coal gangue slurry consists of a crushing system, pulping system, and pumping system. The crushing system is a process equipment system that integrates crushing, screening, and buffering to make gangue into granular aggregate with a specific size distribution through certain technical methods. The pulping system is a process equipment system integrating quantitative feeding, quantitative water supply, and continued mixing to make the crushed particle aggregate into the qualified slurry. The pumping system is a process equipment system integrating pipeline, valve group, monitoring, and control to transport gangue slurry and fill it to the designated goaf by filling pump or gravity flow.

**Figure 3:**
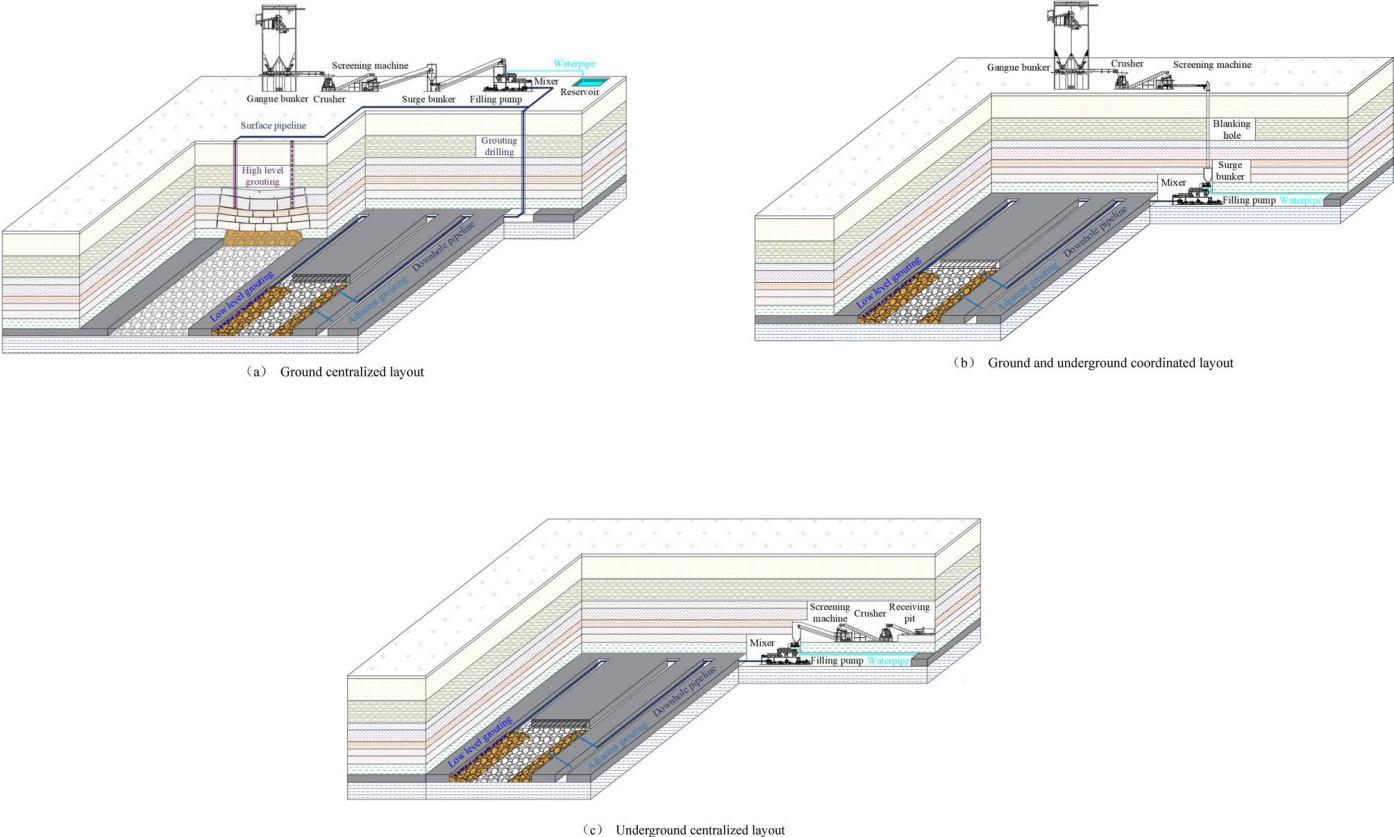
System layout of filling goaf with coal gangue slurry.

Based on the factors such as gangue source, plant location, and roadway layout, the technology system of filling goaf with coal gangue slurry is divided into three types: ground centralized layout, ground and underground coordinated layout, and underground centralized layout, as shown in Fig. 3. The ground centralized layout is to centralize the crushing system, the pulping system, and the power parts of the pumping system at a suitable location on the ground of the mine. The pipeline part of the pumping system is arranged according to the location of the power part and the designated goaf location by selecting a reasonable surface route and underground route to achieve the green disposal of ground washing gangue or gangue dump. The ground and underground coordinated layout are to arrange the crushing and screening equipment of the crushing system at a suitable position on the ground of the mine, arrange the pulping system and the power part of the pumping system at a suitable position underground, select a reasonable underground route for the pipeline part of the pumping system according to the location of the power part and the designated goaf location. However, the blanking hole and surge bunker of the crushing system is used to connect the ground crushing system and the underground pulping system to realize the coordination and matching of ground crushing, underground pumping, and pumping. The underground centralized layout is to centralize the crushing system, the pulping system, and the power parts of the pumping system at the appropriate location of the underground roadways and chambers. The pipeline part of the pumping system is arranged according to the location of the power part and the designated goaf location to select a reasonable underground route to achieve the goal of underground tunneled gangue and sorted gangue without lifting the well.

#### Method of filling goaf with coal gangue slurry

According to the relationship between the pipeline laying path at the end of the pumping system and the location of the target goaf, the method of filling the goaf with coal gangue slurry can be divided into three filling methods: low-level grouting, adjacent-level grouting, and high-level grouting, as shown in Fig. 3. Low-level grouting is a filling method in which pipes are directly arranged in the goaf behind the mining face. Similar to the traditional yellow mud grouting principle of the mine, it mainly fills the small area of the local caving zone behind the mining face with a relatively limited filling amount. Adjacent-level grouting refers to the filling method in which coal gangue slurry is injected into the adjacent goaf during underground drilling. It includes grouting from the main roadway to the mined-out area, grouting from the local mining roadway to the adjacent goaf, grouting from the adjacent mining roadway to the goaf behind the local face, and grouting from the lower coal roadway to the upper coal goaf. It mainly fills most of the caving and fracture zones on both sides of the goaf, with a relatively large filling volume. High-level grouting refers to a grouting filling method in which coal gangue slurry is injected into the goaf by drilling on the ground. It mainly fills the overburden separation zone or water diversion fissure zone, with the most considerable filling.

### The selection process of technology models of filling goaf with coal gangue slurry

According to different filling system forms and filling methods, the technology models of filling goaf with coal gangue slurry can be divided into seven forms: ground centralized pulping + high-level grouting, ground centralized pulping + adjacent-level grouting, ground centralized pulping + low-level grouting, ground and underground coordinated pulping + adjacent-level grouting, ground and underground coordinated pulping + low-level grouting, underground centralized pulping + adjacent-level grouting, and underground centralized pulping + low-level grouting. In addition, the same filling system can be combined with different filling methods to derive multiple technology models of filling goaf with coal gangue slurry.

**Figure 4:**
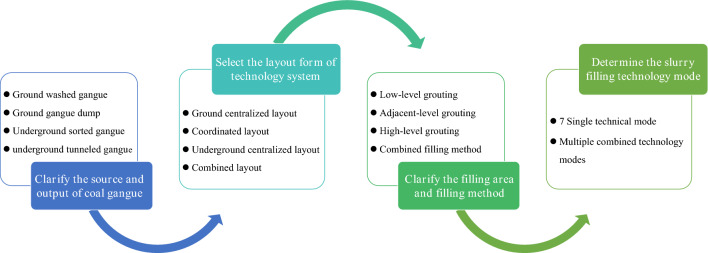
The selection process of technology models of filling goaf with coal gangue slurry.

In field engineering applications, appropriate filling system layout and filling method should be selected according to the source of coal gangue, the ground plant layout, and the underground mining layout of the mine to determine the specific filling technology mode. The selection process of technology models of filling goaf with coal gangue slurry is shown in Fig. 4, specific process: ① Clarify the source and output of coal gangue. The source of coal gangue in the coal mine includes the ground-washed gangue, the ground gangue dump, the underground sorted gangue, and the underground tunneled gangue. ② Select the layout form of the technology system. By analyzing the source location of coal gangue, the comparison between the ground and underground and the underground roadway layout, the appropriate system layout form or combination form is selected from the three forms of the ground centralized layout, ground and underground coordinated layout, and underground centralized layout. When the gangue comes from the ground washed gangue or the ground gangue dump, both the ground centralized layout and the coordinated layout can be selected. A underground centralized layout is generally chosen when the gangue comes from underground sorting or tunneled. ③ Clarify the filling area and filling method. The low-level grouting filling method can only fill the local caving zone behind the mining face. The adjacent-level grouting filling method can fill the goaf's caving zone and fracture zone. Usually, the high-level grouting filling method can fill the overburden separation zone or water-conducting fracture zone. When the system layout is the ground centralized, low-level grouting filling, adjacent-level grouting filling, and high-level grouting filling can all be selected. Generally, only low-level grouting and adjacent grouting filling methods are selected when the system layout is the coordinated or underground centralized layout. ④ Determine the slurry filling technology mode. The appropriate slurry-filling technology mode or combination mode is determined from the seven technology modes by integrating various factors.

## Low-level grouting filling test in Haidaze Coal Mine

### Background of low-level grouting filling test

Dahaize Coal Mine is located west of Yuyang District, Yulin City, Shaanxi Province. The industrial site of the mine and the coal preparation plant are arranged separately and connected by a 3.0 km trestle. The designed production capacity of the mine is 15.0Mt/a, which adopts full vertical shaft development and zonal ventilation, and is equipped with two significant mining height fully mechanized working faces. Dahaize Coal Mine is a new mine. Currently, working face 20101 in panel 201 is being mined, and working face 20201 in panel 201 is being arranged. The sand, gravel, and other materials used for concrete production underground shall be vertically fed through the No. 1 blanking hole and No. 2 blanking hole.

According to the current situation of mine construction, underground mining, and coal preparation plant, the gangue of Dahaizi Coal Mine mainly comes from the tunneling gangue during the capital construction period and the washing gangue of the coal preparation plant, with the amount of washed gangue about 0.7 Mt/a. According to the preliminary design of the mine, all the coal gangue during the construction of the Dahaize Coal Mine is used to lay the special railway subgrade for the mine. Furthermore, the washed gangue is treated with the technology of filling goaf with coal gangue slurry.

### Simulation test of low-level grouting filling

To explore the feasibility of low-level grouting filling and its slurry intervention law, a simulation experiment of low-level grouting filling was conducted in the gangue hill of Dahaize Coal Mine, analyzing the flow and diffusion law of gangue slurry in the simulated goaf during low-level grouting.

#### Simulation model for gangue accumulation in local caving zone

**Figure 5:**
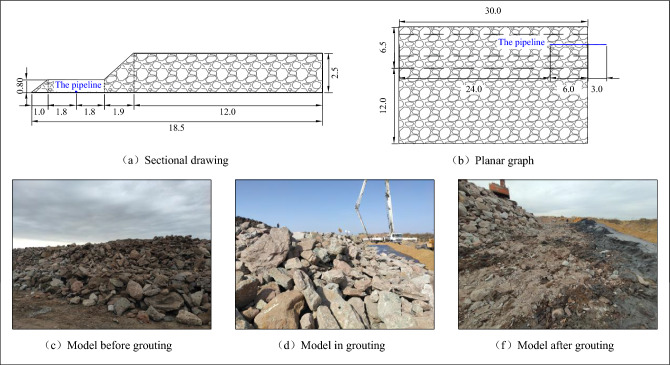
Simulation test model for low-level grouting filling.

Based on the stepped rock block collapse accumulation characteristics in the lower part of the arc-shaped triangular area after the extraction of coal seam 2 in Dahaize Coal Mine and low-level grouting filling area, the rock block collapse morphology of the accumulation caving zone is simulated on the ground. The overall model is stepped, with a size of 30.0 m × 18.5 m × (0.8 –2.5) m, the low-level grouting pipe is laid at the bottom of the model, and the boundary of the model is constrained by sand, as shown in Fig. 5.

#### Simulation test process for low-level grouting filling

Firstly, the gangue from the coal washing plant is crushed into powder with a certain particle size and added with water and additives to produce a 70.0% concentration of gangue slurry at the concrete mixing station, the ratio of gangue, water, and additives is 35:14:1. Secondly, the slurry is continuously transported by concrete tank trucks to the vicinity of the local collapse zone accumulation model of the gangue yard. Finally, the slurry inside the tank trucks is pumped through pipelines to the simulated goaf for grouting and filling.

#### Simulation test analysis of low-level grouting filling

From on-site observation and monitoring data, it can be seen that the flow and diffusion of gangue slurry in the gaps between rock blocks in the simulated caving zone have the following characteristics:

1. The flow of gangue slurry in the gaps between rock blocks is selective. When the size of the gap between rock blocks is greater than 3–4 times the maximum particle size of the accumulated gangue slurry, the gangue slurry is distributed in the gaps between surrounding rock blocks. When the size of the rock block gap is less than 3–4 times the maximum particle size of the accumulated gangue slurry, there is no or a small amount of gangue slurry in the rock block gap near the rock block gap.
2. Starting from the outlet of the grouting pipeline, the overall vertical height of the gangue slurry far from the outlet point shows a downward trend, with the downward trend in the direction of inclination being significantly greater than the downward trend in the direction of strike. The maximum vertical height of the slurry is 2.0 m, and the horizontal distance from the pipeline outlet is 0.4 m. The minimum vertical height of the slurry is 0.75 m, and the horizontal distance from the pipeline outlet is 25.4 m. The gangue slurry is prone to form slurry accumulation points at the dip and strike boundaries, and the maximum accumulation amount is at the intersection of the strike and strike boundaries, as shown in Fig. 6.

**Figure 6:**
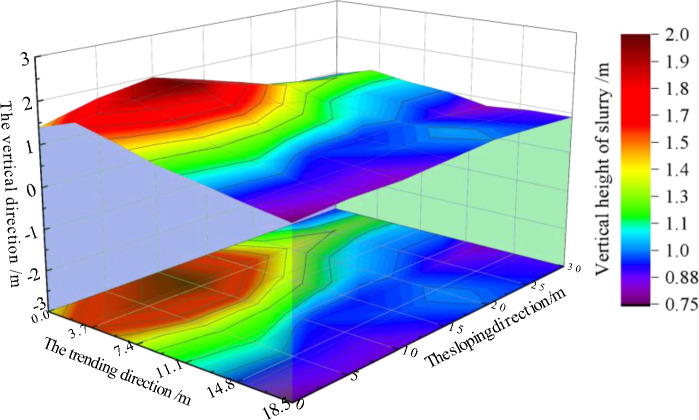
Flow and diffusion law of low-evel grouting filling.
3. Along the flow direction of the gangue slurry, the proportion of coarse particles in the gangue slurry shows a decreasing trend, while the proportion of fine particles shows an increasing trend, the mass fraction of the gangue slurry also shows a decreasing trend. And as the goaf deepens, the proportion of coarse particles in the gangue slurry decreases, and the mass fraction of the gangue slurry also decreases.

### Engineering test of low-level grouting filling

#### Determination of slurry filling model in Dahaize Coal Mine

According to the existing conditions of the mine and the selection process of the slurry filling technology mode, the crushing system, pulping system, and pumping system are respectively arranged on the ground and underground near the No. 1 blanking hole. The low-level grouting filling mode is formed in the goaf behind the 20101 working face in panel 201. Then the ground and underground coordinated pulping and low-level grouting filling are established to explore the reasonable parameters of slurry filling technology of Dahaize Coal Mine and provide data support for the subsequent system expansion and reconstruction.

#### Engineering test process for low-level grouting filling

**Figure 7:**
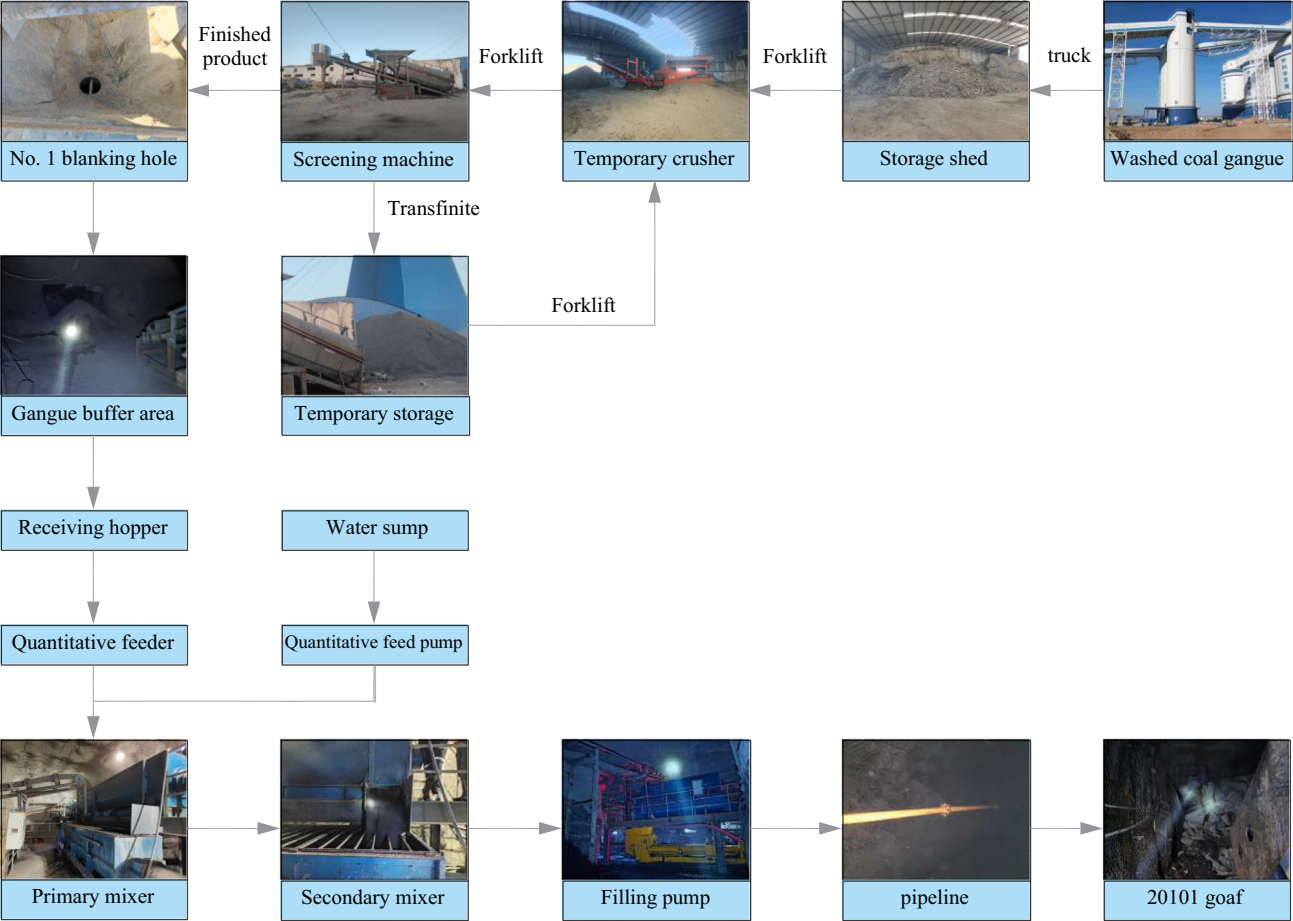
The engineering test process of low-level grouting filling.

Because this test needs to use the forklift to transfer gangue, and the surge buffer has not been set up, its front-end crushing and back-end pulping systems cannot work continuously, so this test process is described by subsystem. Its test process is shown in Fig. 7.

Process of a crushing system: the truck is used to transport the gangue of −50 mm in the gangue bin of the coal washing plant to the gangue boron storage near the No.1 blanking hole of the industrial site. Then the forklift is used to transfer the gangue to the crusher for crushing. The crushed gangue is then transferred to the screening machine by the forklift for screening. The finished particle aggregate under the screen is directly put into the underground buffer through the No.1 blanking hole. The large particle aggregate on the screen is unloaded to the ground. It is transferred to the crusher by forklift for further crushing.

Process of pulping and pumping system: during the filling operation, the forklift transfers the finished particle aggregate buffered at the bottom of the No. 1 blanking hole to the receiving hopper. Then, the quantitative feeder under the receiving hopper and the quantitative feed pump of the clean water silo transport the finished particle aggregate and mine water to the primary mixer at a ratio of 7:3 for pulping, and the qualified, prepared slurry flows automatically to the secondary mixer for buffer mixing. Then it flows automatically to the slurry receiving hopper in the filling pump. Finally, the filling pump will fill the gangue slurry in the slurry receiving hopper to the caving zone of the goaf behind the 20101 working face by low-level grouting through the south wing return air main roadway pipeline and 20101 return air roadway pipeline.

#### Design of engineering test parameters for low-level grouting filling

The filling material of coal gangue slurry is composed of coal gangue and mine water, and the coal gangue is the main aggregate of the filling material. Based on low-level grouting filling simulation test and multiple on-site tests and debugging, this engineering test removed the gangue with a particle size greater than 3 mm, resulting in an average proportion of 26.32% of the 1.25–3 mm particle aggregate, 53.03% of the 0.075–1.25 mm particle aggregate, and 16.74% of the 0–0.075 mm particle aggregate. The ratio of gangue slurry is coal gangue to mine water = 7:3, and the mass fraction of gangue slurry is 70%.

**Figure 8:**
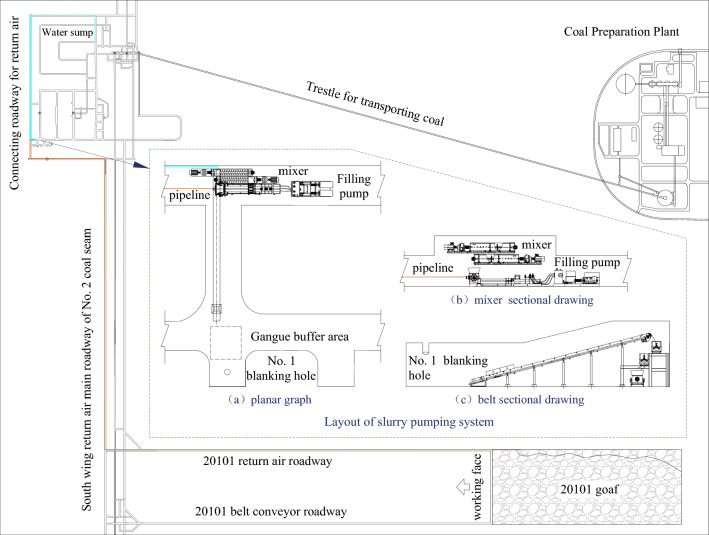
The system layout of low-level grouting filling.

The grouting pump used in this experiment is a plunger pump, with a maximum theoretical delivery capacity of 200m^3^/h at the outlet and a maximum theoretical delivery pressure of 14.0 MPa at the outlet. The underground pipeline model is φ133 × 12 mm, arranged in sequence at the bottom of the north and south wing return air connecting roadway, No.1 return air shaft, south wing return air main roadway, and 20101 belt conveyor roadway, with a total length of approximately 4000 m, as shown in Fig. 8.

Based on the experimental conditions and supporting equipment of this test, the critical flow rate for low level grouting filling in the return air roadway of Dahaize in 20101 was calculated according to the formula of Changsha Research Institute of Mining and Metallurgy:

1 $$V_{c} = 2.809\left( {\frac{{\rho_{s} - \rho_{h} }}{{\rho_{h} }}} \right)^{ - 0.308} C_{V}^{ - 0.308} D^{0.31}$$

In the formula, *V*_C_ is the critical flow rate for slurry transportation, m/s. *ρ*_*S*_ is the density of gangue, kg/m^3^. *ρ*_*H*_ is the density of water, kg/m^3^; *C*_*V*_ is the volume fraction of the gangue slurry. *D* is the inner diameter of the pipeline, m.

After calculation, the critical flow rate for low level grouting filling is 1.71 m/s. Based on 1.1 times the critical flow rate, the design flow rate for slurry transportation should not be less than 1.88 m/s, and the design flow rate should not be less than 63.12m^3^/h.

#### Analysis of engineering test effect of low-level grouting filling

The Dahaize Coal Mine adopted a coordinated arrangement system above and below the shaft and a low level grouting filling method to conduct a coal gangue slurry filling test in the goaf. Without affecting the normal production of the working face, the washed coal gangue was successfully filled into the collapse zone of the underground goaf. In the low-level grouting filling test, the pump pressure of the grouting pump is around 8–10 MPa, and the transportation state of the gangue slurry pipeline is stable. The average transportation flow rate is about 1.94 m/s, and the low-level grouting filling is carried out at an average interval of 30 m. The single filling amount of coal gangue is between 150 and 600 t.

## Adjacent-level grouting filling test in Huangling No. 2 Coal Mine

### Background of adjacent-level grouting filling test

Huangling No. 2 Coal Mine is located in Huangling County, Shaanxi Province, with a production capacity of 10.0 Mt/a. It adopts single horizontal development with an inclined shaft and is equipped with two longwall working faces for mining. Currently, the mining of panel one has been completed, panel 2 is mining working face 211, and panels three and 4 are skip mining. Currently, panel 301 has been completed, and panel 4 is mining working face 420.

During the production period of Huangling No. 2 Coal Mine, the annual output of gangue is about 1.05 million t/a, of which the gangue washed by the coal washing plant is 850,000 t/a, which is mainly used as raw materials for power generation of the gangue power plant. The underground excavated gangue is 200,000 t/a, lifted to the air shaft site through the No.2 air shaft and stacked in the waste dump. With the promotion of ecological and environmental civilization construction and the tightening of national environmental protection policies, the waste dump of Huangling Well 2 has been forbidden to use, and the existing waste dump and its site need to be greened. The underground excavation waste in the later stage must be disposed of harmless. Therefore, Huangling No. 2 Well adopts the technology of filling goaf with coal gangue slurry to treat the underground driving gangue.

### Simulation test of adjacent-level grouting filling

In order to explore the feasibility of adjacent-grouting filling and its slurry intervention law, a simulation test of adjacent-grouting filling was conducted in the laboratory to analyze the flow and diffusion law of gangue slurry in the simulated goaf during adjacent-level grouting.

#### A box model of gangue stacking in local caving zone

**Figure 9:**
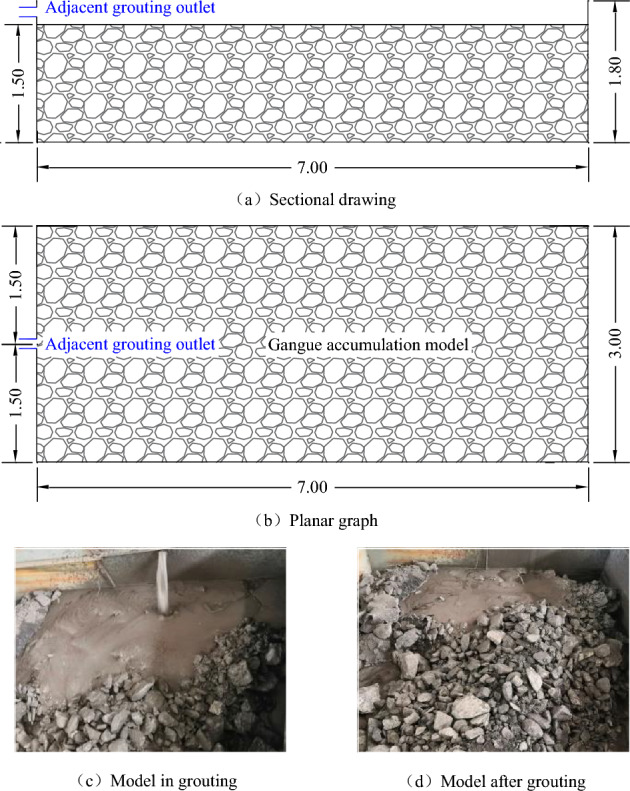
Simulation test model for adjacent grouting filling.

Based on the characteristics of rock block collapse and accumulation in the lower part of the arc-shaped triangular area after mining in the 3rd panel of Huangling No. 2 well, and the adjacent grouting filling area, the rock block collapse morphology of the accumulation and collapse zone was simulated in a large box in the laboratory, with a model size of 3.0 m × 7.0 m × 1.8 m, adjacent-level grouting drilling holes are designed on the left boundary of the model, as shown in Fig. 9.

#### Simulation test process for adjacent-level grouting filling

Firstly,using quantitative feeding equipment, the finished gangue from the storage bin is transported to the mixer through a belt, and is mixed with water in a specific proportion to form a 70% concentration of gangue slurry. The gangue slurry then overflows into the slurry receiving hopper of the grouting pump, and finally the grouting pump pumps the gangue slurry from the slurry receiving hopper through a pipeline to the goaf inside the simulated box for grouting and filling.

#### Simulation test analysis of adjacent-level grouting filling

Based on on-site observation and monitoring data, it can be seen that the flow and diffusion of gangue slurry in the gaps between simulated collapse zone rock blocks during adjacent grouting filling have the following characteristics:

1. As the distance from the adjacent-level grouting port increases, the stacking height of gangue slurry shows a decreasing trend, and due to the constraint of the sloping boundary, the stacking height of gangue slurry in the trending direction decreases significantly faster than in the sloping direction. In the direction of trending, the average self flow slope of gangue slurry within the range of 0–3 m is 18.82%, and the average self flow slope of gangue slurry within the range of 3–7 m is 7.1%, which is 3.8% higher than the calculated self flow slope of gangue slurry collapse, as shown in Fig. 10.

**Figure 10:**
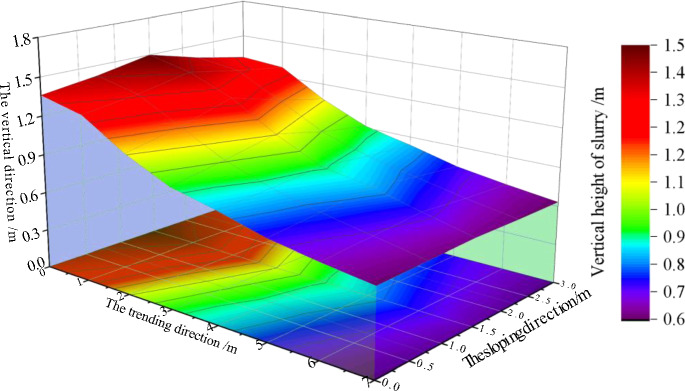
Flow and diffusion law of adjacent-level grouting filling.
2. The flow of gangue slurry between stacked rock blocks is more selective. As it moves away from the adjacent-level grouting port, the proportion of fine particles in the gangue slurry is significantly higher than that of coarse particles. In areas with small gaps in local stacked rock blocks, large particle aggregation and settlement phenomena of 2.0–3.0 mm are observed, that is, when the gaps in stacked rock blocks are less than a certain value, coarse particles in the gangue slurry aggregate and settle, and the flow rate and direction of fine particles change or continue to aggregate and settle in that area.

### Engineering test of adjacent-level grouting filling

#### Determination of slurry filling model in Huangling No. 2 Coal Mine

According to the existing conditions of the mine and the selection process of the slurry filling technology mode, it is proposed to arrange the crushing system near the ground gangue hill. Pulping and pumping systems are arranged near the 301 mined-out area. The adjacent-level grouting filling method is constructed using the 301 mined-out area of the third panel and the 303 return airway (301 auxiliary transport roadway). Then the ground and underground coordinated pulping and adjacent-level grouting filling are established to explore the reasonable parameters of the Huangling No. 2 Coal Mine slurry filling technology and provide data support for the subsequent system expansion and reconstruction.

#### Engineering test process for adjacent-level grouting filling

**Figure 11:**
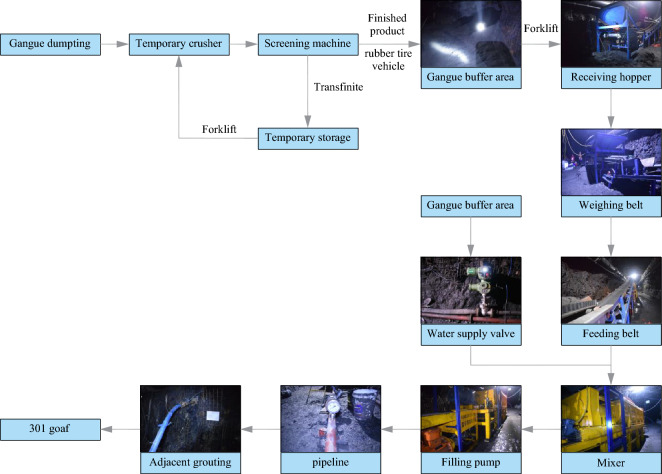
The engineering test process of adjacent-level grouting filling.

As the gangue in this test comes from the ground gangue hill, the crushing system is temporarily arranged near the ground gangue hill, and the pulping system and pumping system are arranged in 301 auxiliary transportation lane (303 return air lane). The test process is shown in Fig. 11.

They used the crushing system to crush the gangue into −3 mm granular aggregate on the ground. Then, use the trackless rubber-tired truck to transport the finished granular aggregate to the underground storage chamber in the 301 auxiliary haulage roadway. The finished granular aggregate is transferred to the receiving hopper by the forklift and enters the mixer through the transfer belt and weighing belt. At the same time, the water supply system supplies quantitative water to the mixer. The mixer will make the finished granular aggregate and water into a uniform slurry. Then the qualified slurry will overflow from the mixer to the receiving hopper of the filling pump. Finally, the grouting pump will fill the gangue slurry to the goaf collapse belt through the transmission pipeline and adjacent grouting holes.

#### Analysis of engineering test effect of adjacent-level grouting filling

The coal gangue slurry filling material is composed of coal gangue and mine water, and the coal gangue is the main aggregate of the filling material. After crushing and screening, the average proportion of particle aggregates with sizes ranging from 1.25 to 3.0 mm is 18.18%, the average proportion of particle aggregates with sizes ranging from 0.075 to 1.25 mm is 65.08%, and the average proportion of particle aggregates with sizes less than 0.075 mm is 16.74%. The ratio of gangue slurry is coal gangue to mine water = 10:7, and the mass fraction of gangue slurry is 70%.

**Figure 12:**
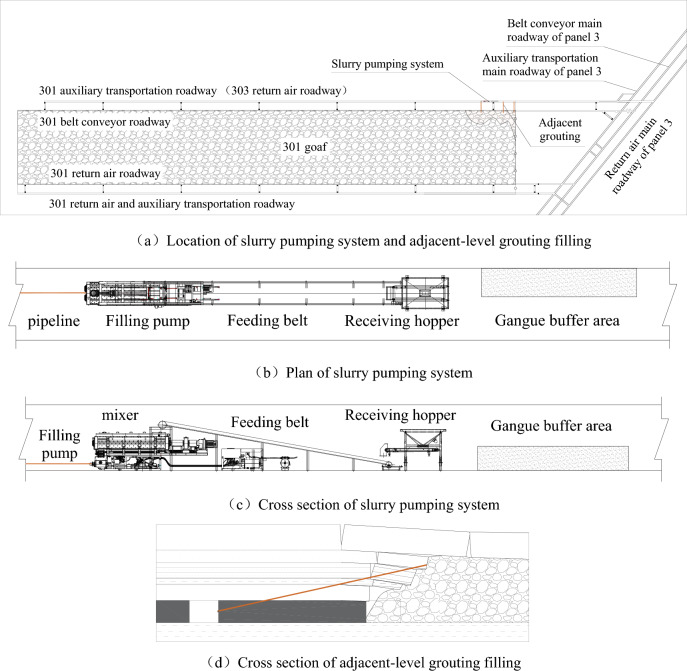
The system layout of adjacent-level grouting filling.

The mixer selected for the experiment is an overflow type, which means that the prepared slurry overflows from the mixer; The filling pump is a plunger pump, with a maximum theoretical delivery capacity of 30m^3^/h at the outlet and a maximum theoretical delivery pressure of 21.0 MPa at the outlet. The underground pipeline is arranged in the 301 auxiliary transportation roadway, near the adjacent-level grouting hole, with an inner diameter of 60 mm and flange connection, as shown in Fig. 12.

Based on the experimental conditions and supporting equipment of this experiment, the critical flow rate during adjacent grouting filling of Huangling No.2 well is calculated according to the formula of Changsha Mining and Metallurgy Research Institute.

By combining formula (1), the critical flow rate for adjacent-level grouting filling is calculated to be 1.31 m/s. If calculated as 1.1 times the critical flow rate, the design flow rate for slurry transportation should not be less than 1.44 m/s and the design flow rate should not be less than 14.65 m^3^/h.

#### Analysis of the experimental effect of adjacent-level grouting engineering

**Figure 13:**
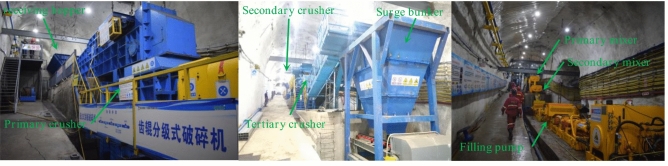
The industrialized slurry filling system of Huangling No. 2 Coal Mine.

After the adjacent-level grouting filling test in Huangling No. 2 Coal Mine, the coal gangue slurry was successfully filled into the caving zone of goaf 301, realizing the green disposal of ground gangue. During the adjacent grouting filling period, the pump pressure of the grouting pump remained stable at around 8.0 MPa, and the transportation status of the gangue slurry pipeline was stable. The average transportation flow rate was about 1.5 m/s, and the single hole grouting volume exceeded 700 m^3^.Huangling No. 2 Coal Mine has re-established the underground centralized pulping + adjacent grouting filling near the bottom gangue bin of the No.2 air shaft and began industrial disposal of underground tunneling gangue, as shown in Fig. 13.

## Conclusions

1. This paper puts forward the technology of filling goaf with coal gangue slurry for green disposal of coal gangue. It gives the principle, technical framework, and technological process of slurry filling technology.
2. Three slurry filling systems are established: ground centralized layout, ground and underground coordinated layout, and underground centralized layout. Three slurry filling methods are established, including low-level grouting, adjacent-level grouting, and high-level grouting. Seven types of technology models are formed, and the selection process of technology models of filling goaf with coal gangue slurry is given.
3. The simulation tests of low level grouting filling and adjacent grouting filling have proven that the gangue slurry can diffuse and flow in the gaps between collapsed rock blocks. The successful application of ground and underground coordinated pulping + low-level grouting and ground and underground coordinated pulping + adjacent-level grouting verified the feasibility of the technology of filling goaf with coal gangue slurry. Moreover, explore the way for the theoretical research of the technology of filling goaf with coal gangue slurry and greening and low interference disposal of coal gangue.

## Data Availability

The datasets used and/or analysed during the current study available from the corresponding author on reasonable request.
